# TREM2 Deficiency Aggravates NLRP3 Inflammasome Activation and Pyroptosis in MPTP-Induced Parkinson’s Disease Mice and LPS-Induced BV2 Cells

**DOI:** 10.1007/s12035-023-03713-0

**Published:** 2023-11-02

**Authors:** Peiting Huang, Zhanyu Zhang, Piao Zhang, Jiezhu Feng, Jianwei Xie, Yinjuan Zheng, Xiaomei Liang, Baoyu Zhu, Zhenzhen Chen, Shujun Feng, Lijuan Wang, Jiahong Lu, Yawei Liu, Yuhu Zhang

**Affiliations:** 1 School of Medicine, South China University of Technology, Guangzhou, 510006 Guangdong Province China; 2 Department of Neurology, Guangdong Neuroscience Institute, Guangdong Provincial People’s Hospital (Guangdong Academy of Medical Sciences), Southern Medical University, Guangzhou, 510080 Guangdong Province China; 3 Guangzhou Key Laboratory of Diagnosis and Treatment for Neurodegenerative Diseases, Guangdong Provincial People’s Hospital (Guangdong Academy of Medical Sciences), Guangzhou, 510080 China; 4 State Key Laboratory of Quality Research in Chinese Medicine, Institute of Chinese Medical Sciences, University of Macau, Macao SAR, China; 5 Department of Neurosurgery & Medical Research Center, Shunde Hospital, Southern Medical University (The First People’s Hospital of Shunde Foshan), Foshan, Guangdong China

**Keywords:** Parkinson’s disease, TREM2, NLRP3, Inflammasome, Pyroptosis

## Abstract

Microglia-mediated neuroinflammation plays a crucial role in the pathogenesis of Parkinson’s disease (PD). Triggering receptor expressed on myeloid cells 2 (TREM2) confers strong neuroprotective effects in PD by regulating the phenotype of microglia. Recent studies suggest that TREM2 regulates high glucose-induced microglial inflammation through the NLRP3 signaling pathway. This study aimed to investigate the effect of TREM2 on NLRP3 inflammasome activation and neuroinflammation in PD. Mice were injected with AAV-TREM2-shRNA into both sides of the substantia nigra using a stereotactic injection method, followed by intraperitoneal injection of MPTP to establish chronic PD mouse model. Behavioral assessments including the pole test and rotarod test were conducted to evaluate the effects of TREM2 deficiency on MPTP-induced motor dysfunction. Immunohistochemistry of TREM2 and tyrosine hydroxylase (TH), immunohistochemistry and immunofluorescence Iba1, Western blot of NLRP3 inflammasome and its downstream inflammatory factors IL-1β and IL-18, and the key pyroptosis factors GSDMD and GSDMD-N were performed to explore the effect of TREM2 on NLRP3 inflammasome and neuroinflammation. In an in vitro experiment, lentivirus was used to interfere with the expression of TREM2 in BV2 microglia, and then lipopolysaccharide (LPS) and adenopterin nucleoside triphosphate (ATP) were used to stimulate inflammation to construct a cellular inflammation model. The expression differences of NLRP3 inflammasome and its components were detected by qPCR and Western blot. In vivo, TREM2 knockdown aggravated the loss of dopaminergic neuron and the decline of motor function. After TREM2 knockdown, the number of activated microglia was significantly increased, and the expression of cleaved caspase-1, NLRP3 inflammasome, IL-1β, GSDMD, and GSDMD-N was increased. In vitro, TREM2 knockdown aggravated the inflammatory response of BV2 cells stimulated by LPS and promoted the activation of NLRP3 inflammasome through the NF-κB pathway. In addition, TREM2 knockdown also promoted the expression of TLR4/MyD88, an upstream factor of the NF-κB pathway. Our vivo and vitro data showed that TREM2 knockdown promoted NLRP3 inflammasome activation and downstream inflammatory response, promoted pyroptosis, and aggravated dopaminergic neuron loss. TREM2 acts as an anti-inflammatory in PD through the TLR4/MyD88/NF-κB pathway, which extends previous findings and supports the notion that TREM2 ameliorates neuroinflammation in PD.

## Introduction

Parkinson’s disease (PD) is a common neurodegenerative disease in the elderly [1, 2], and its main clinical manifestations include bradykinesia, resting tremor, and myotonia, which seriously affect the quality of life of PD patients [3]. The neuropathology of PD is characterized by the deposition of misfolded protein aggregates composed mainly of α-synuclein (α-syn) in different brain regions and the progressive degeneration of dopaminergic neurons in the substantia nigra compacta (SNc), followed by striatal dopamine depletion [4–6]. Recent studies have shown that neuroinflammatory processes and the mechanism of neuroinflammation-mediated neuronal death might play a crucial role in the pathogenesis of PD [7]. Neuroinflammation is a basic immune response that protects neurons from injury and compensates for neuronal damage, but excessive neuroinflammation can exacerbate neuronal damage [8–11]. It is yet unknown how neuroinflammation contributes to the development and progression of PD.

Triggering receptor expressed on myeloid cells 2 (TREM2) is crucial for the microglia-mediated inflammation [12–17]. TREM2, a member of immunoglobulin superfamily receptors, is found on a variety of myeloid cells, such as microglia in the central nervous system. It regulates neuroinflammation, proliferation, survival, activation, and phagocytosis of microglia [18–21]. Numerous studies have shown that TREM2 plays an essential role in microglia-mediated neuroinflammation [12–15]. Guo et al. [22] reported that suppression of TREM2 expression induced the microglia to a pro-inflammatory state, aggravated α-syn-induced inflammatory response in BV2 microglia, and exacerbated the loss of dopaminergic neurons in PD mice. Additionally, our earlier research [23] also revealed that TREM2 could induce the transformation of microglia into M2-type microglia with an anti-inflammatory effect in PD, suggesting that TREM2 plays an inhibitory effect on neuroinflammation in the pathogenesis of PD. Uncertainty still exists regarding the precise mechanism by how TREM2 regulates neuroinflammation in PD.

Numerous studies indicated that during the pathogenesis of PD, there was an enhanced activation of the NOD-like receptor protein 3 (NLRP3) inflammasome, which aggravated neuroinflammation, damaged dopaminergic neurons, and eventually promoted the development of PD [24–30]. Wnting et al. [31] discovered that TREM2 inhibited caspase-1-dependent pyroptosis and a strong inflammatory response in a study of *Pseudomonas aeruginosa* keratitis, indicating that TREM2 may regulate the inflammatory response in *Pseudomonas aeruginosa* keratitis via regulating the NLRP3 inflammasome. So far, there is currently no evidence available regarding the effects of TREM2 on the NLRP3 inflammasome and the pyroptosis in PD.

We therefore knocked down TREM2 expression in an MPTP-induced chronic PD mouse models and a cellular model of classical neuroinflammation to investigate the regulatory relationship between TREM2 and the NLRP3 inflammasome in PD. We discovered that TREM2 deficiency promoted activation of microglia, aggravated NLRP3 inflammasome activation and pyroptosis, and exacerbated the loss of substantia nigra dopaminergic neurons. TREM2 acts as an anti-inflammatory in PD through the TLR4/MyD88/NF-κB pathway.

## Method

### Experimental Animal

C57BL/6 mice (male, 6–8 months old) were obtained from South China University of Technology Animal Core. Mice were maintained and bred in the animal facility at South China University of Technology Animal Centre. All animals were maintained with free access to pellet food and water under specific pathogen-free conditions. Animal welfare and experimental procedures were performed in accordance with the Guide for the Care and Use of Laboratory Animals. Mice were divided into 5 groups according to the randomized grouping method: (1) sham operation group: phosphate buffer saline (PBS) injection using stereotaxic injection technique, followed by intraperitoneal injection of saline; (2) negative control + saline group (NC + saline group): negative control adeno-associated virus (AAV) injected using stereotaxic injection technique, followed by saline injected intraperitoneally; (3) negative control + MPTP group (NC + MPTP group): negative control AAV injected using stereotaxic injection technique, followed by intraperitoneal injection of MPTP; (4) TREM2-knockdown + saline group (KD + saline group): TREM2-interfering adeno-associated virus (AAV-TREM2-shRNA) injected using stereotaxic injection technique, followed by intraperitoneal injection of saline; and (5) TREM2-knockdown + MPTP group (KD + MPTP group): AAV-TREM2-shRNA using the stereotaxic injection technique, followed by intraperitoneal injection of MPTP.

### Stereotaxic Surgery and Treatment of the MPTP-Induced PD Model

The AAV-TREM2-shRNA was injected into the both sides of substantia nigra of the mouse using stereotactic injection method. The AAV-TREM2-shRNA and the corresponding negative control virus were synthesized by Hanheng Biotechnology (Shanghai) Co. The mice were anesthetized by intraperitoneal injection (0.2 ml/10 g) with 0.2% sodium pentobarbital in saline as solvent and fixed on a stereotaxic instrument after anesthesia. Localization of substantia nigra in the midbrain was based on mouse brain tissue atlas. The microinjector was aspirated with a sufficient amount of viral fluid and injected at a rate of 0.2 µl/min, waited for 5 min, and then slowly withdrawn. MPTP intraperitoneal injection was performed after the mice had healed (2 weeks). The mice were injected with 1.0 µl of adeno-associated virus at the rate of 0.2 µl/min, with the coordinates at AP − 3.28 mm, ML ± 1.25 mm, and DV + 4.6 mm relative to the bregma. The needle was left in place for 5 min before being retracted. MPTP intraperitoneal injection was performed when the mice had healed (2 weeks later). Mice were injected intraperitoneally with 25 mg/kg of MPTP solution (2.5 mg/ml) or an equivalent volume of saline on the left side of the body at 3.5-day intervals for a total of 10 injections. All mice were subjected to behavioral tests 5 days after the last injection.

### Animal Behavioral Assessment

#### Traction Test

The front paws of the mice were placed on a horizontal rope with a diameter of 5 mm, and the suspension of the limbs of the mice was observed after 10 s. Mice with two rear paws grasping the rope scored 4 points; mice with one rear paw grasping the rope scored 3 points; mice with two front paws grasping the rope scored 2 points, and animals with one front paw grasping the rope scored 1 point.

#### Rotarod Test

A 30-min training test was conducted every day 3 days before the start of the experiment. In the formal experiment, the mouse was placed on the rod, with its back to the rotation direction, so that it had to walk forward to stay on the rod. The start speed is adjusted to 3 rpm, and the acceleration rate is set to 3 rpm/min. The maximum speed is 20 rpm. Start the motor and timer. When the mouse falls off the rod, the test ends. Record the latency from rotating the rod to falling and the rotation speed of the mouse when falling. Repeat the above process 3 times.

### Brain Tissue Sampling

At the end of the behavioral tests, mice were anesthetized by sodium pentobarbital (40 mg/kg b.w., i.p.). For qPCR and western blotting analysis, the whole brains were rapidly extracted from animals, and then the midbrain samples were quickly dissected and pre-frozen by liquid nitrogen. All samples were stored at − 80 °C until analysis. For immunohistochemical (IHC) analysis, mice were perfused transcardially with 4% paraformaldehyde (PFA). Brains were extracted, post-fixed, dehydrated, and embedded in paraffin. The lateral ventricle and the third ventricle were located according to the mouse brain atlas to determine the location of the substantia nigra of the midbrain. The midbrain was sliced with a thickness of 3 µm and placed in the oven at 60 °C.

### Cell Culture and Treatment

The immortalized murine microglial cell line BV2 was maintained at 37 °C in a 5% CO_2_ humidified incubator in DMEM supplemented with 10% fetal bovine serum and 50 U/ml penicillin and streptomycin. Select BV2 cells with good growth, aspirate the original culture medium, wash with PBS and digest with trypsin, add fresh culture medium to terminate digestion, centrifuge the supernatant, add fresh culture medium to make cell suspension, and take a small amount of cell suspension on the counting plate for cell counting. The cells were diluted with fresh complete medium to 5 × 10^4^ cells/ml, and the diluted cell suspension was absorbed into a six-well plate with 2 ml per well, and placed into an incubator and cultured at 37 °C in 5% CO_2_ environment.

The TREM2-interfering lentivirus (TREM2-shRNA) and the corresponding negative control virus were synthesized by Gekko Gein Medical Technology Co. LTD (Shanghai). TREM2-shRNA consisted of the following sequence: AGAAGCGGAATGGGAGCA and CTCGGAGACTCTGACACTGGTA. After 24 h, the original medium was removed; fresh medium, appropriate amount of virus solution, and auxiliary dye were added, for a total of 1 ml per well. The transfected virus was an empty virus and a shTREM2 lentivirus, and it was then cultured in an incubator. After about 16 h of culture, the cells were observed, and the fresh medium was changed for further culture. About 72 h after infection, the efficiency of infection was observed using fluorescence microscope. BV2 cells were observed to grow well after infection, and 1 µg/ml puromycin was added for cell screening. After 5 days of screening, the fresh complete medium was changed to allow the surviving cells to proliferate, pass, and freeze for subsequent experiments.

BV2 microglia with good growth after lentivirus infection were selected, digested, and resuspended, and the cells were counted. The cells were seeded into six-well plates at 2 × 10^5^ cells/well, mixed, and cultured in an incubator for 24 h. One microgram per milliliter of lipopolysaccharide (LPS) and 1 mM of adenopterin nucleoside triphosphate (ATP) as inflammatory stimulants were used and then divided into the scramble-TREM2 group, TREM2-shRNA group, scramble-TREM2 + LPS + ATP group, and TREM2-shRNA + LPS + ATP group; the corresponding stimulants were added to each group, cultured in the incubator, and treated for 12 h, and the treated cells were collected for subsequent experiments.

### Western Blot

Total protein was lysated with RIPA protein extraction buffer containing protease inhibitors and phosphatase inhibitor mixture, and then the protein concentrations were quantified using a BCA Kit. Samples were separated through SDS-PAGE and transferred to polyvinylidene fluoride (PVDF) membranes. The samples were blocked with 5% BSA dissolved in Tris-buffered saline with 0.1% Tween 20 (TBST) at room temperature (RT) for 1 h. Then, the membranes were incubated with primary antibodies overnight at 4 °C and subsequently incubated with HRP-conjugated secondary antibodies at RT for 1 h. The target protein signal was detected and digitized using ECL solution and Image J program. Analyses were completed in three experiments, and the mean value was calculated.

### Real-Time PCR Quantification

Total RNA was extracted from BV2 cells by using TRIzol regent and subjected to cDNA synthesis. Then, quantitative real-time PCR was performed by SYBR Premix Ex Taq (Takara). The sequences of the specific primers for target genes are listed below. β-actin: forward (5′-GTGACGTTGACATCCGTAAAGA-3′) and reverse (5′-GCCGGACTCATCGTACTCC-3′); TREM2: forward (5′-AGAAGCGGAATGGGAGCA-3′) and reverse (5′-CTCGGAGACTCTGACACTGGTA-3′); NLRP3: forward (5′-ATTACCCGCCCGAGAAAGG-3′) and reverse (5′-CATGAGTGTGGCTAGATCCAAG-3′); IL-1β: forward (5′-GAAATGCCACCTTTTGACAGTG-3′) and reverse (5′-TGGATGCTCTCATCAGGACAG-3′); IL-18: forward (5′-GACTCTTGCGTCAACTTCAAGG-3′) and reverse (5′-CAGGCTGTCTTTTGTCAACGA-3′); TLR4: forward (5′-GCCATCATTATGAGTGCCAAT-3′) and reverse (5′-AGGGATAAGAACGCTGAGAATT-3′); and MyD88: forward (5′-CACCTGTAAAGGCTTCTCG-3′) and reverse (5′-CCCACTCGCAGTTTGTTG-3′). The data of real-time PCR were analyzed using the value 2^−ΔΔCt^. β-catin was used as the internal control.

### Immunohistochemistry

The paraffin sections were dewaxed and hydrated for thermal antigen repair, and the sections were blocked with 3% hydrogen peroxide. Primary antibodies were incubated at 4 °C overnight and then treated with fluorescent or horseradish peroxidase–labeled secondary antibodies. After DAB color development, it was stained with hematoxylin, and after sealing the film, it was photographed with an upright microscope.

### Immunofluorescence

Paraffin sections were dewaxed and hydrated before thermal antigen repair, and sections were blocked with 5% BSA. The primary antibody was incubated overnight at 4 °C; the secondary antibody fluorescent antibody was added for 1 h, and DAPI was used for nuclear counterstaining. The samples were then imaged using a fluorescence microscope.

### Enzyme-Linked Immunosorbent Assay (ELISA)

The cells of each group were removed from the incubator, and the cell supernatant was aspirated in 1.5-ml EP tubes, followed by centrifugation at 2000 PRM for 20 min to remove impurities and cell debris. The supernatant was removed into a new 1.5-ml EP tube for subsequent assays. An aliquot of each supernatant was assayed in duplicate for IL-1β protein by using ELISA kits from Lunchang Biotechnology Co., LTD (Xiamen) following the manufacturer’s instructions.

### Statistical Analysis

All experiments were performed with at least three independent replicates. The obtained data values were presented as the mean ± SEM (standard error of the mean). The data were analyzed by one-way ANOVA to compare the various groups. The Mann–Whitney test was used to compare the continuous variables that did not satisfy the normality test. Differences were considered statistically significant when the corrected *P* value was < 0.05. All statistical analysis was performed using GraphPad Prism 7.0 and SPSS statistical software package version 20.0.

## Result

### TREM2 Knockdown Aggravated the Loss of Dopaminergic Neuron the Decline of Motor Function in MPTP-Induced PD Mice

**Fig. 1:**
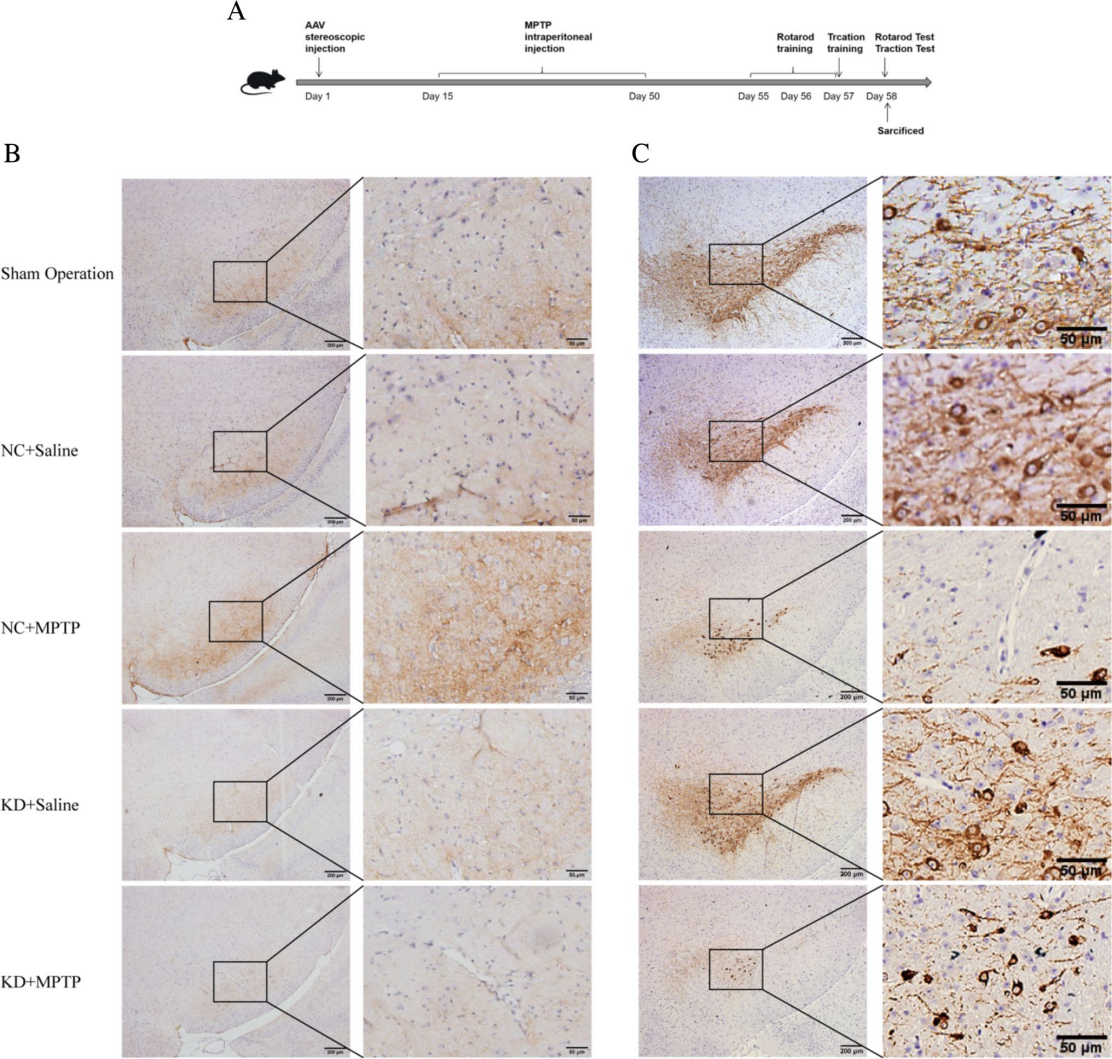

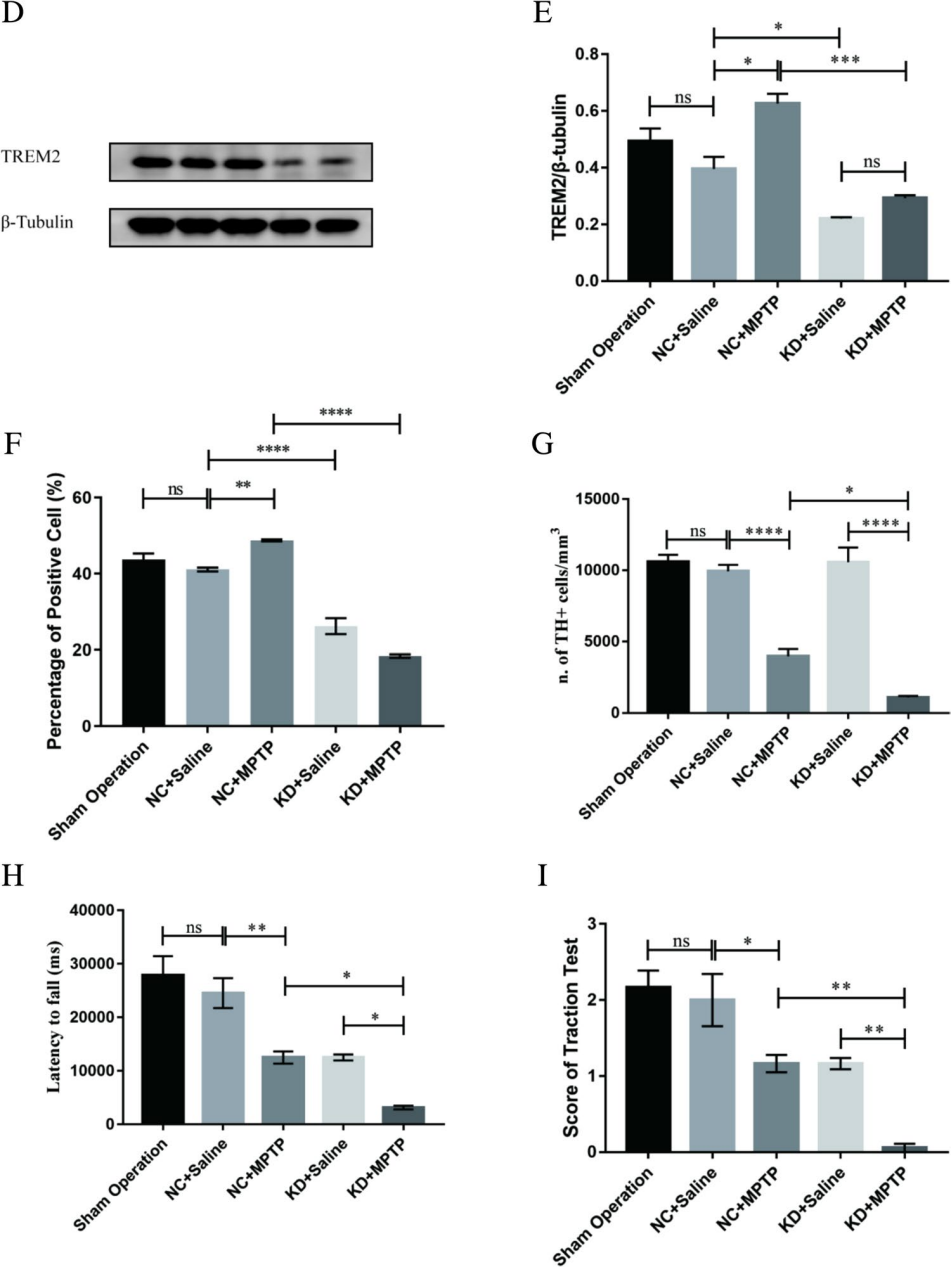
TREM2 knockdown aggravated the loss of dopaminergic neuron the decline of motor function in MPTP-induced PD mice. **A** The scheme of experimental procedure. **B** Immunohistochemical staining of TREM2 in brain substantia nigra (SN) from indicated mice. Scale bar = 200 µm and 50 µm. **C** Immunohistochemical staining of TH in brain SN from indicated mice. Scale bar = 200 µm and 50 µm. **D** Western blotting analysis of TREM2 from indicated mice (*n* = 3/group). **E** Data are shown as representative plot and quantified immunoblotting bands. **F** Quantified cell numbers of TREM2 positive cells in SN. **G** Number of TH cells in SN measured by stereological counting. **H** Latency to fall during test on accelerated rotarod for 3 consecutive trials was recorded and analyzed. **I** The average scores in the traction test. Data are representative of the results of three independent experiments (mean ± SEM). Significant differences are indicated as follows: *P < 0.05, **P < 0.01, ***P < 0.001, and ****P < 0.0001

In this study, C57BL/6 mice were injected with AAV-TREM2-shRNA to knock down the expression of TREM2 in the substantia nigra of the midbrain by stereotactic injection technique, and then chronic PD mice were established by intraperitoneal injection of MPTP and probenecid. One week after modeling, the motor function of mice was evaluated by the traction test and rotarod test (Fig. 1A). To determine the knockdown efficiency of AAV-TREM2-shRNA in each group and the expression of TREM2 in the substantia nigra of the midbrain of mice, we measured the level of TREM2 in the substantia nigra of the midbrain of mice by immunohistochemical staining (Fig. 1B, F). After the injection of AAV-TREM2-shRNA using the stereotaxic injection technique, the TREM2 expression level was significantly decreased in the KD + saline group and the KD + MPTP group. Meanwhile, the protein level of TREM2 was measured by Western blot (Fig. 1D, E). Consistent with the results of immunohistochemical staining, the level of TREM2 protein decreased significantly after knockdown of TREM2 expression in the KD + saline group and the KD + MPTP group, indicating that the model of TREM2 knockdown was successfully established. As shown in Fig. 1C and G, the number of TH-positive cells in the MPTP-induced PD mouse model decreased significantly compared with the control group, and the number of TH-positive cells decreased further after knocking down TREM2 expression, indicating that the model of MPTP-induced PD mice was successfully established, and the knockdown of TREM2 aggravated the loss of dopaminergic neurons. In the rotarod test (Fig. 1H), NC + MPTP mice spent less time moving on the rotarod than NC + saline mice, and KD + MPTP mice also spent less time moving on the rotarod than NC + MPTP mice. Similarly, In the traction test (Fig. 1I), the scores of mice in the NC + MPTP group were lower than those in the NC + saline group, and the scores of mice in the KD + MPTP group were significantly lower than those in the NC + MPTP group. These results indicated that the decrease of TREM2 expression aggravated the decline of motor function in MPTP-induced PD mouse.

### TREM2 Knockdown Promoted Microglial Activation in MPTP-Induced PD Mice

**Fig. 2:**
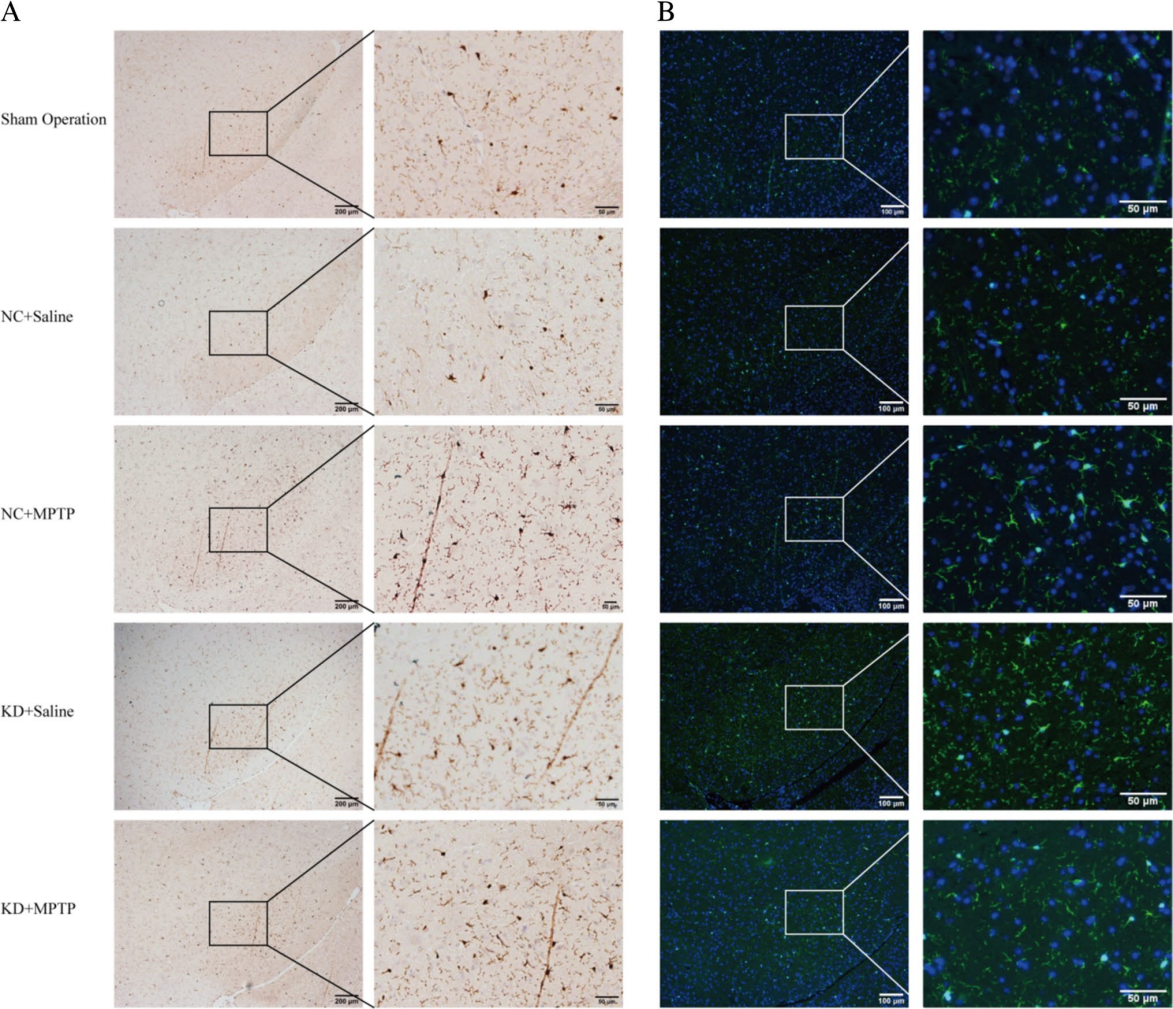

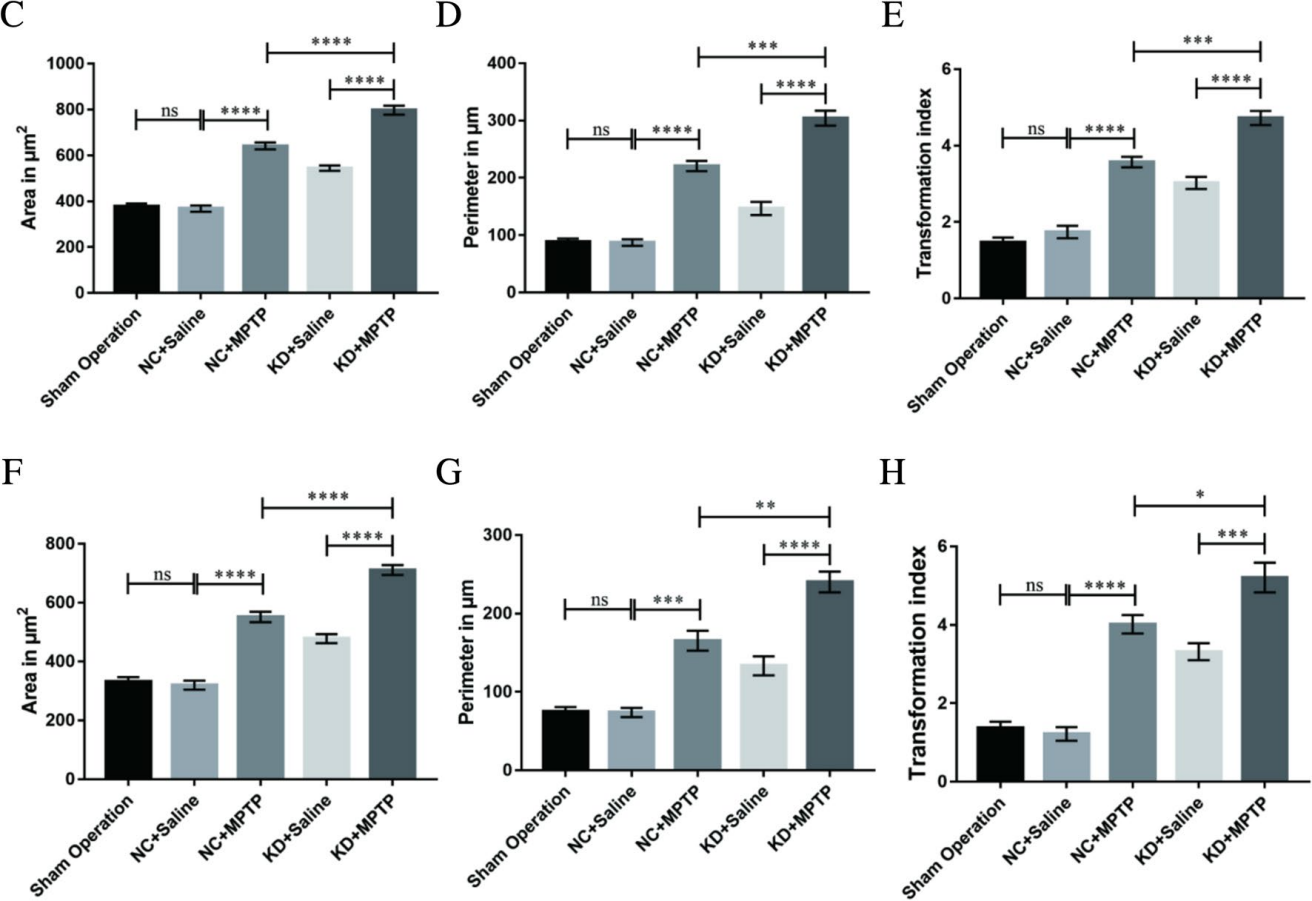
TREM2 knockdown promoted microglial activation in MPTP-induced PD mice. **A** Immunohistochemical staining of ionized calcium binding adapter molecule 1 (Iba1) in SN from indicated mice. Scale bar = 200 µm and 50 µm. **B** Immunofluorescence analysis of microglia Iba1 (green) in brain SN from indicated mice. Scale bar = 100 µm and 50 µm. Quantified surface area (**C**, **F**) in µm.^2^, perimeter (**D**, **G**) in µm, and TI values (**E**, **H**) of the microglia in SN for immunohistochemical staining (**C**, **D**, **E**) and immunofluorescence staining (**F**, **G**, **H**). Data are representative of the results of three independent experiments (mean ± SEM). Significant differences are indicated as follows: *P < 0.05, **P < 0.01, ***P < 0.001, and ****P < 0.0001

Studies have shown that microglia activation is a core process of neuroinflammation and an important factor in the pathogenesis of PD. Therefore, we observed the activation of microglia by immunohistochemical staining (Fig. 2A) and immunofluorescence staining (Fig. 2B). The quantitative analysis was based on the area, perimeter, and TI. TI is an indicator for the degree of process extension of a cell. Throughout the experiments, microglial cells with TI < 3 were considered ameboid. In immunohistochemical staining (Fig. 2A, C–E), the unchallenged, untreated microglia culture consisted mainly of ameboid cells with an average area of 377.08 ± 27.25 µm^2^, perimeter of 88.90 ± 10.55 µm, and a TI of 1.18 ± 0.25. When treated with MPTP, the microglial became enlarged and acquired a significantly larger perimeter and TI (area = 641.32 ± 29.78 µm^2^, perimeter = 220.62 ± 17.51 µm, TI = 3.7 ± 0.28). Interestingly, when TREM2 knocked down, the cell surface area, perimeter, and TI were significantly increased compared with the NC + MPTP group (area = 797.78 ± 39.02 µm^2^, perimeter = 304.15 ± 26.23 µm, TI = 4.64 ± 0.37), which indicated microglia was activated. In immunofluorescence staining (Fig. 2B, F–H), the microglia also became enlarged and acquired significantly larger perimeter and TI in the KD + MPTP group (area = 710.66 ± 34.42 µm^2^, perimeter = 240.05 ± 26.41 µm, TI = 5.21 ± 0.76). Both immunohistochemical and immunofluorescence results showed that TREM2 knockdown significantly increased the area, perimeter, and transformation index of microglial, namely, TREM2 knockdown promoted microglial activation.

### TREM2 Deficiency Aggravates NLRP3 Inflammasome Activation and Pyroptosis in MPTP-Induced PD Mice

**Fig. 3:**
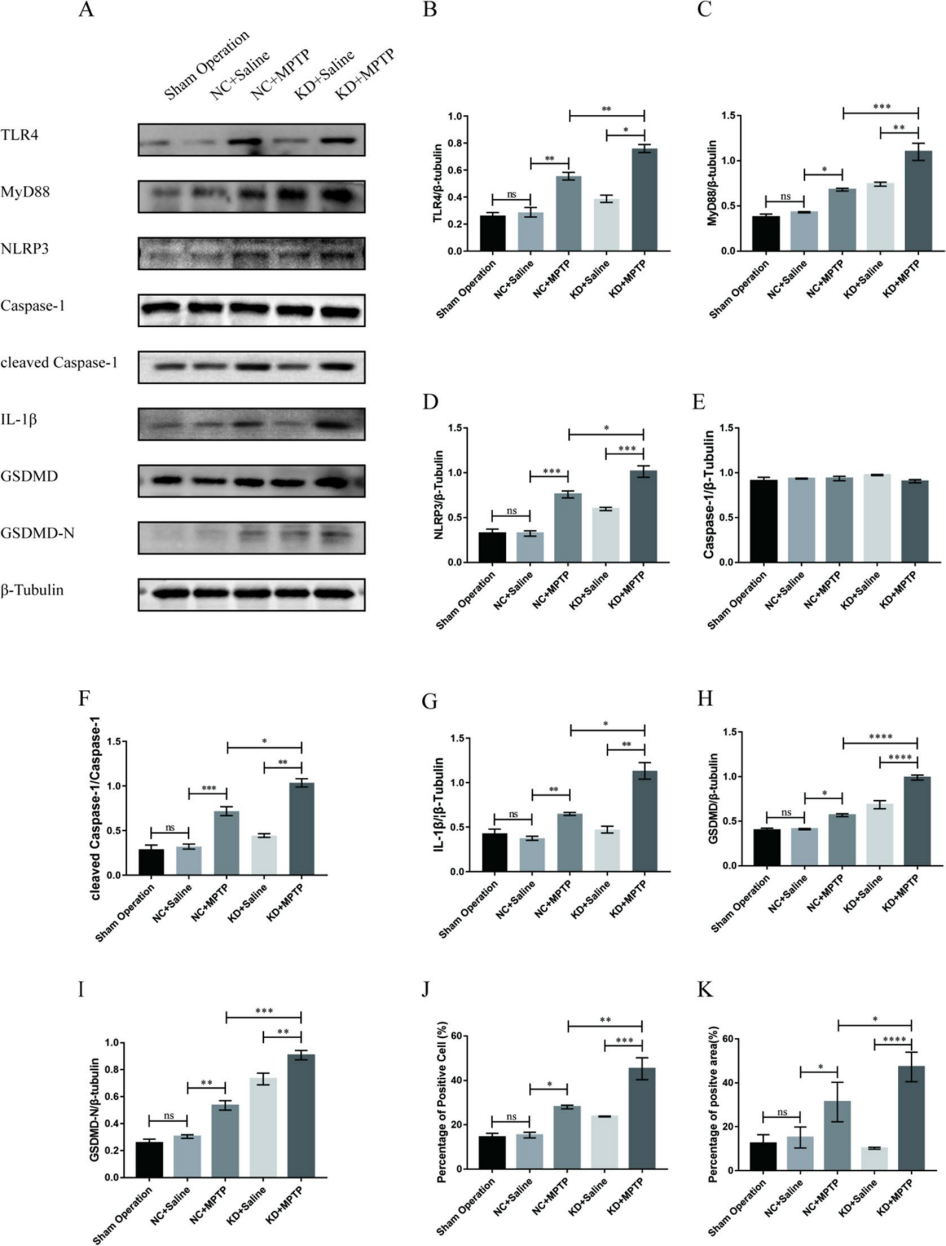

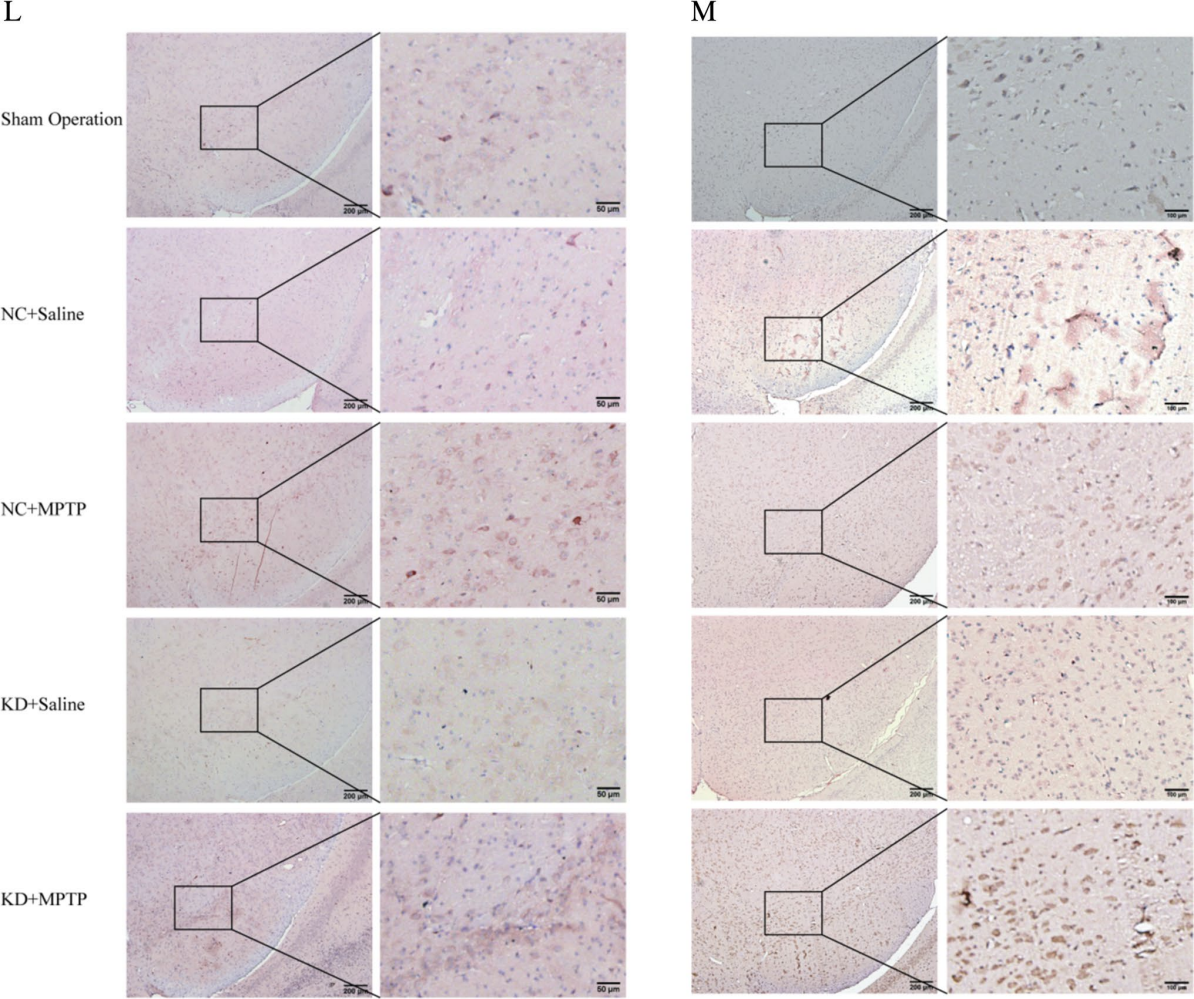
TREM2 deficiency aggravates NLRP3 inflammasome activation and pyroptosis in MPTP-induced PD mice. **A**–**K** Western blotting analysis of TLR4, MyD88, NLRP3, caspase-1, cleaved caspase-1, IL-1β, GSDMD, and GSDMD-N in the mesencephalon tissue from indicated mice (*n* = 3/group). Data are shown as **A** representative plots and **B**–**I** quantified immunoblotting bands. **J** Quantified the percantage of cleaved caspase-1 positive cells in SN. **K** Quantified the percantage of IL-18 positive cells in SN. **L** Immunohistochemical staining of cleaved caspase-1 in brain SN from indicated mice. Scale bar = 200 µm and 50 µm. **M** Immunofluorescence analysis of IL-18 in brain SN from indicated mice. Scale bar = 200 µm and 50 µm. Data are representative of the results of three independent experiments (mean ± SEM). Significant differences are indicated as follows: *P < 0.05, **P < 0.01, ***P < 0.001, and ****P < 0.0001

Our previous study found that silencing TREM2 expression promoted microglia-mediated inflammatory response; conversely, overexpression of TREM2 inhibited microglia-mediated inflammatory responses and produced anti-inflammatory factors, suggesting a protective role of TREM2 in PD neuroinflammation. Therefore, to investigate the mechanism by which TREM2 suppresses PD neuroinflammation, we examined the expression levels of NLRP3 inflammasome and its downstream inflammatory factors in each group of mice. As shown in Fig. 3A–K, the protein expression levels of NLRP3 inflammasome, its activated effector protein cleaved caspase-1, and the downstream key inflammatory factor IL-1β were increased in the NC + MPTP group compared with the control group, indicating that NLRP3 inflammasome is significantly activated in the process of PD, which aggravates neuroinflammation, thereby leading to the damage of dopaminergic neurons. However, after TREM2 was knocked down, the protein levels of NLRP3 inflammasome, IL-1β, GSDMD, and GSDMD-N in the KD + MPTP group were significantly higher than those in the NC + MPTP group, indicating that the reduction of TREM2 expression can lead to increased activation of NLRP3 inflammasome and aggravate pyroptosis in PD mice, thereby aggravating neuroinflammation. Immunohistochemical staining (Fig. 3L, M) showed that the expression levels of cleaved caspase-1 and IL-18 in the substantia nigra of the midbrain of mice in the TREM2 knockdown group were significantly higher than those in the NC + MPTP group, indicating that TREM2 knockdown promoted the activation of NLRP3 inflammasome and downstream inflammatory response at the in situ level. At the same time, we detected the protein expression levels of TLR4 and MyD88, which are upstream of NLRP3 inflammasome. As shown in Fig. 3B and C, the protein expression levels of TLR4 and MyD88, which are upstream priming signal molecule of NLRP3 inflammasome, were significantly increased after TREM2 knockdown compared with the NC + MPTP group. These results suggest that TREM2 may regulate NLRP3 inflammasome through TLR4/MyD88 pathway.

### Knockdown of TREM2 Promoted the Activation of NLRP3 Inflammasome and the Level of Inflammation and Pyroptosis in LPS-Stimulated BV2 Cells

To determine the appropriate LPS stimulation concentration, BV2 microglia cells were treated with 0.01 µg/ml, 0.1 µg/ml, 1 µg/ml LPS, and 1 mM ATP for 12 h. Western blot was used to detect NLRP3 protein. The expression of NLRP3 protein in BV2 microglia cells increased, and the expression of NLRP3 inflammasome protein increased most significantly after the treatment of BV2 microglia cells with 1 µg/ml LPS. Therefore, 1 µg/l of LPS was selected for subsequent experiments. In order to determine the optimal concentration of ATP, BV2 microglia cells were treated with 1 mM, 1.5 mM, 2 mM, 2.5 mM, and 3 mM of ATP and 1 µg/ml LPS for 12 h, and NLRP3 inflammasome was detected by Western blot. It was found that after LPS + ATP treatment, the protein expression of NLRP3 inflammasome in BV2 microglia changed to different degrees. When the ATP concentration was 1 mM, 1.5 mM, and 2 mM, the expression of NLRP3 protein increased, but there was no significant difference, while when the ATP concentration was 2.5 mM and 3 mM, the expression of NLRP3 protein decreased. Therefore, ATP concentration of 1 mM was selected for subsequent experiments in this study.

**Fig. 4:**
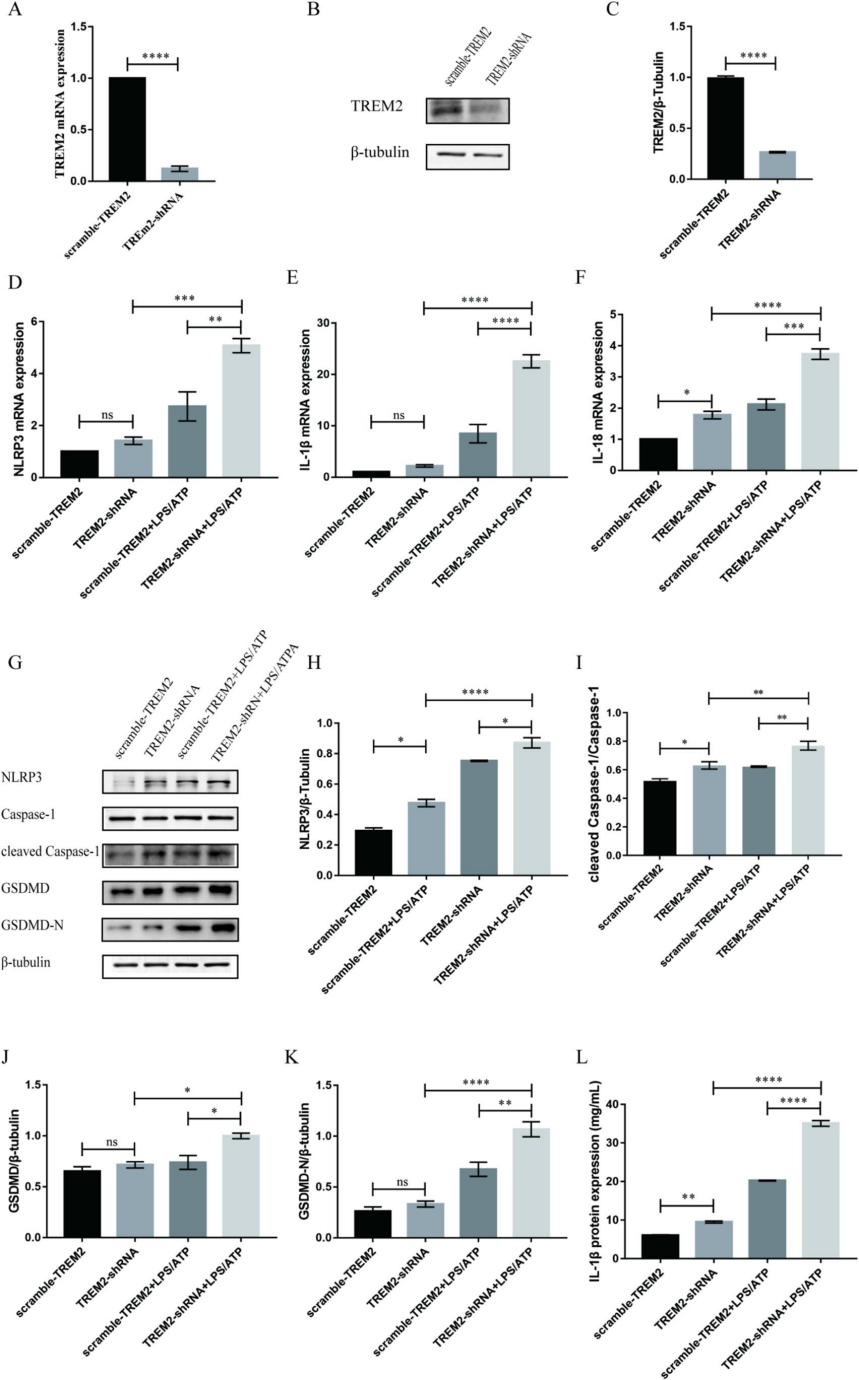
Knockdown of TREM2 promoted the activation of NLRP3 inflammasome and increased the levels of its downstream inflammatory factors in LPS-stimulated BV2 cells. **A** TREM2 transcription level after stimulation with LPS + ATP (LPS: 1 µg/ml, ATP: 1 mM) in BV2 cells as detected by qPCR. **B**–**C** Western blot analysis of TREM2 in the scramble-TREM2 group and TREM2-shRNA group (*n* = 3/group). β-tubulin was used as an internal control for normalization. Data are shown as **B** representative plots and **C** quantified Western blotting bands. **D**–**F** Transcription level of NLRP3, IL-1β, and IL-18 after stimulation with LPS + ATP (LPS: 1 µg/ml, ATP: 1 mM) in BV2 cells as detected by qPCR. **G**–**K** Western blotting analysis of NLRP3, caspase-1, cleaved caspase-1, GSDMD, GSDMD-N in WT control, and TREM2-KO BV2 cells (*n* = 3/group). β-tubulin was used as an internal control for normalization. Data are shown as **G** representative plots and **H**–**K** quantified Western blotting bands. **L** ELISA detection of IL-1β in BV2 cells treated with LPS + ATP (LPS: 1 µg/ml, ATP: 1 mM). Data are representative of the results of three independent experiments (mean ± SEM). Significant differences are indicated as follows: *P < 0.05, **P < 0.01, ***P < 0.001, and ****P < 0.0001

To construct a BV2 cell model with TREM2 knockdown, BV2 microglia cells were infected with TREM2-shRNA lentivirus (negative control adopts scramble-TREM2 lentivirus) to interfere TREM2 expression. After 72 h of infection, the effect of lentivirus infection was observed by fluorescence microscope, and the interference efficiency of TREM2 was detected by fluorescence quantitative PCR and Western blot. As shown in Fig. 4A–C, after using TREM2-shRNA lentivirus to interfere with BV2 microglia cells, TREM2 mRNA and protein levels were significantly decreased, with the knockdown efficiency reached 93%, suggesting that TREM2 expression was successfully knocked down in BV2 cells, which could be used in subsequent cell experiments.

Next, LPS + ATP (LPS: 1 µg/ml, ATP: 1 mm) were used to induce the activation of NLRP3 inflammasome in BV2 microglia cells, which is the classic inflammatory stimulation model of BV2. To further investigate the activation of NLRP3 inflammasome and pyroptosis factors in microglia cells, qPCR, Western blot, and ELISA were used to detect the expression of NLRP3, caspase-1, cleaved caspase-1, IL-1β, GSDMD, and GSDMD-N. As shown in Fig. 4D–F, the mRNA levels of NLRP3, IL-1β, and IL-18 in the TREM2-shRNA group were higher than those in the scramble-TREM2 group. After LPS + ATP stimulation, the mRNA levels of NLRP3, IL-1β, and IL-18were further increasing in the TREM2-shRNA + LPS/ATP group. Secondly, Western blot was used to detect NLRP3 inflammasome, cleaved caspase-1, GSDMD, and GSDMD-N protein expression (Fig. 4G–K). The results showed that the protein expression levels of NLRP3 inflammasome, cleaved caspase-1, GSDMD, and GSDMD-N were increased after LPS + ATP stimulation. The expression of NLRP3, cleaved caspase-1, GSDMD, and GSDMD-N in the TREM2-shRNA + LPS/ATP group was significantly higher than those in the scramble-TREM2 + LPS/ATP group. The similar result also occurred in the ELISA detection of IL-1β (Fig. 4L). The results showed that after LPS + ATP treatment, the expression of IL-1β in the two groups was significantly increased. However, the expression of IL-1β in the TREM2-shRNA group increased more significantly than in the scramble-TREM2 group. These results suggest that reduced TREM2 expression promotes NLRP3 inflammasome activation and the level of inflammation and pyroptosis in LPS-stimulated BV2 cells.

### Knockdown of TREM2 Promoted the Expression of TLR4/MyD88 Pathway and Promoted the Activation of NLRP3 Inflammasome Through NF-κB in LPS-Stimulated BV2 Cells

**Fig. 5:**
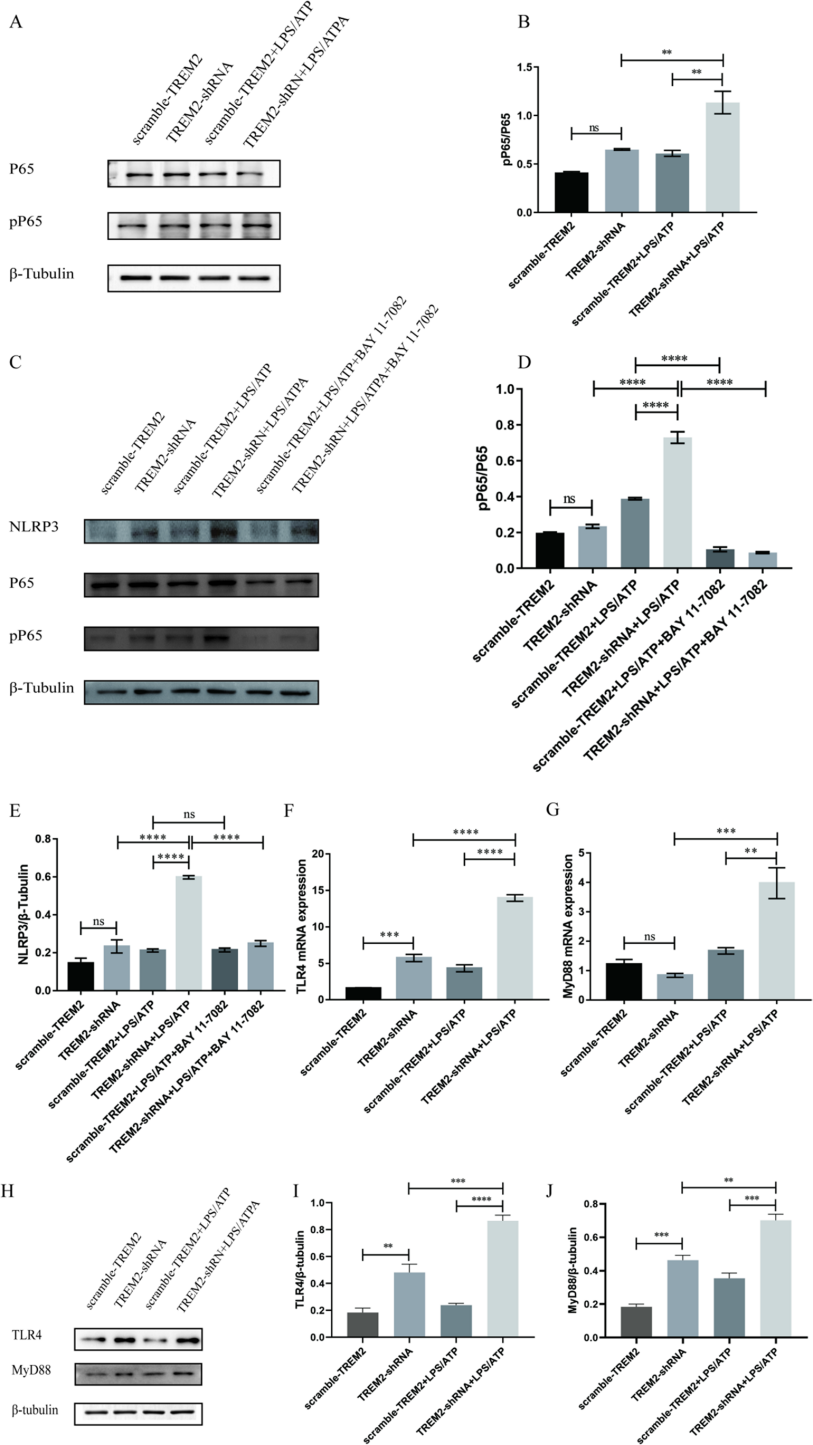
Knockdown of TREM2 promoted the expression of TLR4/MyD88 pathway and promoted the activation of NLRP3 inflammasome through NF-κB in LPS-stimulated BV2 cells. **A**–**B** Western blot analysis of P65 and pP65 in BV2 cells treated with LPS + ATP (LPS: 1 µg/ml, ATP: 1 mM) (*n* = 3/group). β-tubulin was used as an internal control for normalization. Data are shown as **A** representative plots and **B** quantified Western blotting bands. **C**–**E** Western blot analysis of P65, pP65, and NLRP3 in BV2 cells treated with BAY7082-11 (*n* = 3/group). β-tubulin was used as an internal control for normalization. Data are shown as **C** representative plots and **D**–**E** quantified Western blotting bands. **F**–**G** ELISA detection of TLR4 and MyD88 in BV2 cells treated with LPS + ATP (LPS: 1 µg/ml, ATP: 1 mM). **H**–**J** Western blot analysis of TLR4 and MyD88 in BV2 cells treated with LPS + ATP (LPS: 1 µg/ml, ATP: 1 mM) (*n* = 3/group). β-tubulin was used as an internal control for normalization. Data are shown as **H** representative plots and **I**–**J** quantified Western blotting bands. Data are representative of the results of three independent experiments (mean ± SEM). Significant differences are indicated as follows: *P < 0.05, **P < 0.01, ***P < 0.001, and ****P < 0.0001

Previous studies have shown that the NF-κB pathway is a key pathway for NLRP3 inflammasome activation. Therefore, we examined the effect of reduced TREM2 expression on the phosphorylation of P65, a key molecule of the NF-κB pathway. Western blot detection of the key molecule P65 and phosphorylation P65 (pP65) of NF-κB pathway showed that the phosphorylation P65 in the TREM2-shRNA group was significantly higher than that of the scramble-TREM2 group, and after LPS + ATP stimulation, the expression of pP65 in the TREM2-shRNA group increased more significantly than that in the TREM2-shRNA group (Fig. 5A–B). To further verify whether TREM2 down-regulates NLRP3 inflammasome by inhibiting NF-κB pathway activation, we pretreated NF-κB pathway inhibitor bay7082-11 (10-µm pretreatment for 30 min) and then detected the protein levels of pP65 and NLRP3 inflammasome by Western blot. As shown in Fig. 5C–E, in the group pretreated with NF-κB pathway inhibitor bay7082-11, the expression of pP65 and NLRP3 inflammasome was significantly decreased compared with the control group without bay7082-11. These results suggest that TREM2 regulates NLRP3 inflammasome expression through the NF-κB pathway in LPS-treated BV2 cells.

It has been reported that the NF-κB pathway is regulated by the toll-like receptor pathway. Therefore, to investigate whether TREM2 inhibits the NF-κB pathway by inhibiting the toll-like receptor signaling pathway, we examined the expression of TLR4 receptor and its downstream adaptor molecule MyD88 in BV2 microglia after TREM2 expression reduction. Consistent with in vivo results, the mRNA and protein expression levels of TLR4 and MyD88 (Fig. 5F–J), which are upstream priming signal molecule of NLRP3 inflammasome, were significantly increased after TREM2 knockdown in the TREM2-shRNA + LPS/ATP group. The above results show that knockdown of TREM2 promoted the mRNA and protein levels of TLR4/MyD88 pathway and increased the expression of NLRP3 inflammasome through NF-κB in LPS-stimulated BV2 cells.

## Discussion

Numerous studies have demonstrated that TREM2 is crucial for the microglia-mediated neuroinflammation [12–15, 22]. Multiple vitro and vivo studies support the anti-inflammatory function of TREM2, which has been classically described as an anti-inflammatory factor [14]. TREM2 inhibited NF-κB and MAPK signaling activation mediated by toll-like receptor4 (TLR4) and tumor necrosis factor receptor-associated factor 6 (TRAF6) in MPTP-induced mice PD models, reducing MPTP-induced neuropathic changes [17]. Additionally, conditioned media from TREM2-siRNA transfected BV2 microglia-induced apoptosis of cultured SH-SY5Y cells by inhibiting TREM2 expression and increasing pro-inflammatory factor expressions [22]. However, TREM2 can mediate or increase inflammatory responses, according to numerous additional research. For instance, TREM2 knockdown reduced ROS production [32, 33]. Pro-inflammatory cytokine levels were also decreased in TREM2 deficient mice after traumatic brain injury [34], ischemia [35], lung infection [36], and demyelination [37], where TREM2 deficient brain myeloid cells showed a less activated morphology [20]. The above researches shown that TREM2 can play a variety of functions in the inflammatory response, depending on the particular stimuli, the strength and duration over which they are presented, the cell type, and the context [14]. The specific mechanism by which TREM2 regulates neuroinflammation in PD is currently unknown. Numerous studies showed that NLRP3 inflammasome activation occurred during the etiology of PD, aggravating neuroinflammation, promoting NLRP3-dependent pyroptosis, damaging dopaminergic neurons, and ultimately promoting the development of PD [24–30, 38–40]. Therefore, using stereotactic injection of AAV-TREM2-shRNA to knock down TREM2 expression in the substantia nigra of the midbrain and intraperitoneal injection of MPTP to establish a chronic PD mouse model, we found that TREM2 knockdown significantly increased the expression of NLRP3 inflammasome and its downstream inflammatory factors IL-1β and IL-18 and the key pyroptosis factors GSDMD and GSDMD-N. This strongly implies that TREM2 contributes to the neuroprotective and anti-inflammatory properties of the PD mouse model, which is consistent with our previous study [23] and the results of Guo et al. [22]. More significantly, our research revealed that TREM2 inhibits pyroptosis, which provided a neuroprotetive effect in PD.

Additionally, in our study, TREM2 knockdown in PD mouse models not only aggravated the activation of NLRP3 inflammasome and pyroptosis but also markedly elevated the expressions of TLR4 and MyD88. According to the studies that have demonstrated that after the binding of LPS and its receptor [41, 42], the first signal of NLRP3 inflammasome activation is initiated by TLR4 and delivered by its mediator molecules, including MyD88, IRAK1, and IRAK4, leading to the NF-κB-dependent transcription of NLRP3 and pro-IL-1β [43–45]. In addition, TREM2 down-regulates TLR4-mediated NF-κB activation and cytokine TNF-α production. Therefore, we make the assumption that TREM2 influences the TLR4/MyD88/NF-κB pathway, which in turn regulates NLRP3 inflammasome activation and pyroptosis. We modeled BV2 cells using the classical inflammasome stimulators LPS and ATP to verify this. Reduced TREM2 expression led to promote the activation of NLRP3 inflammasome, the expression of TLR4 and MyD88, and the phosphorylation of P65, a key protein in the NF-κB pathway, which is in line with our animal model. Treatment with the NF-κB inhibitor BAY7082-11 inhibited the increase in NLRP3 inflammasome by decreased TREM2 expression. These results in our study imply that TREM2 influences the TLR4/MyD88/NF-κB pathway and then inhibit the activation of NLRP3 inflammasome.

Our vivo and vitro research indicates that TREM2 may firstly inhibit the activation of NF-κB pathway by suppressing the expression of TLR4 and MyD88, and then inhibit the activation of NLRP3 inflammasome and its downstream inflammatory cascade and pyroptosis, and finally mitigating the neuroinflammatory and the damage to dopaminergic neurons in PD.

There are certain drawbacks in our study. Our study was conducted only in the TREM2 knockdown model. This means that our research still needs to be further investigated with TREM2 overexpression model. In addition, TREM2 not only inhibits neuroinflammatory responses, but also mediates microglial phagocytosis, lipid metabolism, and energy metabolism. In order to generate fresh ideas for the novel therapeutics for PD, it will be important to further clarify the precise function and mechanism of TREM2-mediated microglia in the degenerative process of PD.

## Data Availability

The datasets generated during and/or analyzed during the current study are not publicly available but are available from the corresponding author on reasonable request.
